# Fat content, energy value and fatty acid profile of donkey milk during lactation and implications for human nutrition

**DOI:** 10.1186/1476-511X-11-113

**Published:** 2012-09-11

**Authors:** Giovanni Martemucci, Angela Gabriella D’Alessandro

**Affiliations:** 1 Department of Agricultural and Environmental Science, University of Bari, Via G. Amendola, 165/A, 70126, Bari, Italy

**Keywords:** Donkey’s milk, Energetic value, Fatty acids, Nutritional remarks

## Abstract

**Background and aims:**

Milk contains numerous nutrients. The content of n-3 fatty acids, the n-6/n-3 ratio, and short- and medium-chain fatty acids may promote positive health effects. In Western societies, cow’s milk fat is perceived as a risk factor for health because it is a source of a high fraction of saturated fatty acids. Recently, there has been increasing interest in donkey’s milk. In this work, the fat and energetic value and acidic composition of donkey’s milk, with reference to human nutrition, and their variations during lactation, were investigated. We also discuss the implications of the acidic profile of donkey’s milk on human nutrition.

**Methods:**

Individual milk samples from lactating jennies were collected 15, 30, 45, 60, 90, 120, 150, 180 and 210days after foaling, for the analysis of fat, proteins and lactose, which was achieved using an infrared milk analyser, and fatty acids composition by gas chromatography.

**Results:**

The donkey’s milk was characterised by low fat and energetic (1719.2kJ·kg^-1^) values, a high polyunsaturated fatty acids (PUFA) content of mainly α-linolenic acid (ALA) and linoleic acid (LA), a low n-6 to n-3 FA ratio or LA/ALA ratio, and advantageous values of atherogenic and thrombogenic indices. Among the minor PUFA, docosahesaenoic (DHA), eicosapentanoic (EPA), and arachidonic (AA) acids were present in very small amounts (<1%). In addition, the AA/EPA ratio was low (0.18). The fat and energetic values decreased (P < 0.01) during lactation.

The fatty acid patterns were affected by the lactation stage and showed a decrease (P < 0.01) in saturated fatty acids content and an increase (P < 0.01) in the unsaturated fatty acids content. The n-6 to n-3 ratio and the LA/ALA ratio were approximately 2:1, with values <1 during the last period of lactation, suggesting the more optimal use of milk during this period.

**Conclusions:**

The high level of unsaturated/saturated fatty acids and PUFA-n3 content and the low n-6/n-3 ratio suggest the use of donkey’s milk as a functional food for human nutrition and its potential utilisation for infant nutrition as well as adult diets, particular for the elderly.

## Background

The relationship between diet and health is one of the keys for preventing disease and promoting well-being. There is an increasing awareness of the role of dietary lipids in health maintenance and disease prevention. Evidence suggests that a high intake of saturated fatty acids (SFA) from the diet may be associated with elevated cardiovascular disease risk [1], and the association between diet and health is a decisive factor in consumer food selection. In addition to the reduction of fat intake, a change in the saturated/unsaturated fatty acid ratio may contribute to improved human health [2].

A European study on fatty acid consumption reported that milk and dairy products are great sources of SFAs [3].

Cow milk possesses poor polyunsaturated fatty acid (PUFA) levels [4]. Donkey’s milk appears to contain lower SFA amounts and higher essential fatty acid (EFA) amounts than cow’s milk, although it appears to feature limited amounts of long-chain PUFA; overall these findings suggest that donkey’s milk is more like human milk than is cow’s milk [5].

Recently, there has been increasing interest in donkey’s milk due to its potential role in human nutrition and especially in paediatric dietetics [6-8].

The determination of the characteristics of donkey’s milk is important for economic interest in human diets and pharmaceuticals. Moreover, the milk composition is affected by the stage of lactation [9,10].

Few studies on the characterisation of the lipid content and energetic value of donkey’s milk [11,12] or on the variation in milk fat content, energetic value and fatty acid composition during lactation have been reported [11,13]. Thus, a deeper knowledge of the fat and energetic composition of this milk is necessary to understand the physiological features of milk, particularly the fatty acid composition, which is of great importance from a nutritional standpoint [14].

The aim of this study was to investigate the fat and energetic value and acidic composition of donkey’s milk over seven months of lactation, and several parameters that could be of interest to human nutrition were highlighted.

## Methods

The experiment was conducted according to protocols approved by the Italian Minister for Scientific Research in accordance with EC regulations.

### Milk samples and analyses

Milk samples were collected during an entire lactation period of seven months (210 days) from nine healthy pluriparous jennies of the Martina Franca breed, aged from 6 to 12 year, with good body conditions (average weight = 320 ± 30 kg; the average body condition was 3.3 ± 0.2 on a scale of 0 to 5 [15]). The animals were reared under the semi-extensive conditions commonly used in the typical breeding area (south-eastern Murgia, Apulia region, southern Italy) (40°37’20” latitude; 60°17’44” longitude), based on natural grazing (7 to 10h of pasture in one grazing session on natural scrub of *Ligustrum vulgare* L., *Arbustus unedo* L., *Pistacia Lentiscus*, *Quercus ilex* L., *Fraxinus ornu* and *Cistus* s.p.) with added oats (2 times per day; 2.0 to 2.5 kg grain· d^-1^· jenny^-1^) and water *ad libitum*. The animals were selected according to a close foaling date (± 7 days). During the lactation period the foals were with their dams and were separated from them before milking (3 h). The asses were milked twice a day, in the presence of foals placed in an adjacent box to allow visual contact, using a mechanical milker with a vacuum level of 42 kPa, a pulse ratio of 50% and a pulse rate of 120 cycles/min [16].

Individual milk samples, from morning and evening milkings, were collected 15, 30, 45, 60, 90, 120, 150, 180, and 210 days after foaling. Milk from the two daily milkings was pooled and split into two aliquots for the analyses of fat, protein and lactose content and for the determination of fatty acid composition. The milk samples were then preserved (−20°C) until analysis.

The fat, protein and lactose contents were determined using an infrared milk analyser (Milkoscan 6000), previously standardised for donkey’s milk according to FIL-IDF 141C [17] to determine the gross energetic value. The gross energetic value of milk, expressed in kJ·kg^–1^, was calculated by the Perrin [18] formula, which considers a coefficient of 9.11 for the fat percentage, 5.86 for the protein percentage and 3.95 for the lactose percentage.

The fatty acids composition of the collected milk samples was determined using capillary gas chromatography. The fat content of milk was extracted with chloroform-methanol (2:1, vol/vol) using the method described by Folch et al. [19]. The fatty acid composition in milk was estimated using methyl esters prepared by direct transesterification, according to IUPAC method 2.301 [20]. The analyses were performed using a Fison HRGC Mega 2 series gas chromatograph (Milan, Italy) with a flame-ionisation detector fitted with WCOT fused-silica capillary column (FFAP-CB coating, 25 m × 0.32 mm i.d. × 0.30 μm film thickness, Chrompack, Middleburg, The Netherlands) using the analytical conditions used by Caponio et al. [21]. The separation was performed at pre-programmed temperatures: 50°C for 3 min; 50–100°C at a rate of 20°C/min; 100°C for 2 min; 100–240°C at a rate of 20°C/min; 240°C for 15 min. Hydrogen was the carrier gas (flow rate, 2mL/min). The injector temperature was 270°C (splitting ratio, 1:17) and the detector temperature was 300°C. Fatty acid peaks were identified using a comparative analysis with standard reference mixtures. The fatty acid content is expressed as percentage of the total identified fatty acids.

The desaturase index was calculated for three pairs of fatty acids representing the products and substrates for Δ^9^-desaturase as follows: *cis*-9 14:1/14:0, *cis*-9 16:1/16:0, *cis*-9 18:1/18:0. For example the desaturase index for *cis*-9 18:1 was (*cis*-9 18:1)/(*cis*-9 18:1+18:0). The atherogenic (AI) and thrombogenic (TI) indices were calculated using the Ulbricht and Southgate [22] equations.

### Statistical analysis

Changes over time in the fat, energetic values and classes or the individual fatty acids of milk from the jennies were analysed by PROC MIXED for repeated measurements of SAS [23]. A GLM procedure was used. The fixed effect included the stage of lactation which was divided into 8 controls considering post-foaling days (d 15, 30, 60, 90, 120, 150, 180 and 210) for the fat composition and gross energy content of milk and into 9 controls (d 15, 30, 45, 60, 90, 120, 150, 180 and 210) for the fatty acid composition, with the residual error being individual within a given control of lactation. The differences among the means were compared using Student’s t tests. The results were considered significant when P ≤ 0.05.

## Results

### Milk fat content and gross energy

The average percentage of milk fat is provided in Table 1 and was low (an average of 0.54%). The highest value occurred at 15 and 30 days with a gradual decreasing trend throughout the lactation period, becoming significant (P < 0.05) at 210 days.

**Table 1:** Average fat (%, w/w) and gross energy content of milk during the lactation period (210 d)

**Lactation stage**	**Fat X ± SD**	**Gross Energy (kJ·kg**^**-1**^**)**
15 d	0.72 ± 0.09 ^a^	1857.3 ^a^
30 d	0.71 ± 0.10 ^a^	1831.5 ^a^
60 d	0.65 ± 0.15	1780.9
90 d	0.54 ± 0.15	1749.6
120 d	0.55 ± 0.17	1764.1
150 d	0.50 ± 0.22	1690.6
180 d	0.48 ± 0.06	1668.4
210 d	0.42 ± 0.33 ^b^	1645.6 ^b^

^a, b ^Means with different letters within the column differ significantly for P < 0.05.

The gross energetic value of donkey’s milk on average was 1748.5 kJ·kg^-1^, with a significant (P < 0.05) decrease (− 10%) from day 15 to 210 (Table 1).

### Fatty acid composition

The acid classes of fat milk were statistically different (P < 0.05) and were influenced by the lactation stage (P<0.01). Overall, the average SFA content was higher than the monounsaturated fatty acid (MUFA) and PUFA content (Table 2).

**Table 2:** Summarized data of milk fatty acid composition (X ± SD)

**Fatty acid composition**	
Saturated (SFA, %)	51.98 ± 5.13
Unsaturated (UFA, %)	48.02 ± 2.97
Monounsaturated (MUFA, %)	28.00 ± 3.91
Polyunsaturated (PUFA, %)	20.02 ± 2.04
of which	
PUFA n-3 (%)	7.12 ± 1.96
PUFA n-6 (%)	12.90 ± 2.13
PUFA n-3/n-6 ratio	0.59 ± 0.08
UFA/SFA ratio	0.92 ± 0.07
Desaturase index^1^	
cis-9 14:1	0.04 ± 0.01
cis-9 16:1	0.14 ± 0.03
cis-9 18:1	0.93 ± 0.01
Atherogenic index (AI)^2^	1.16 ± 0.28
Thrombogenic index (TI)^3^	0.70 ± 0.14

^1^Ratio of Δ^9^-desaturase product divided by the sum of the Δ^9^-desaturase product and product.

^2, 3^Calculated using the equation described by Ulbricht and Southgate (1991).

### Saturated fatty acids

**Figure 1:**
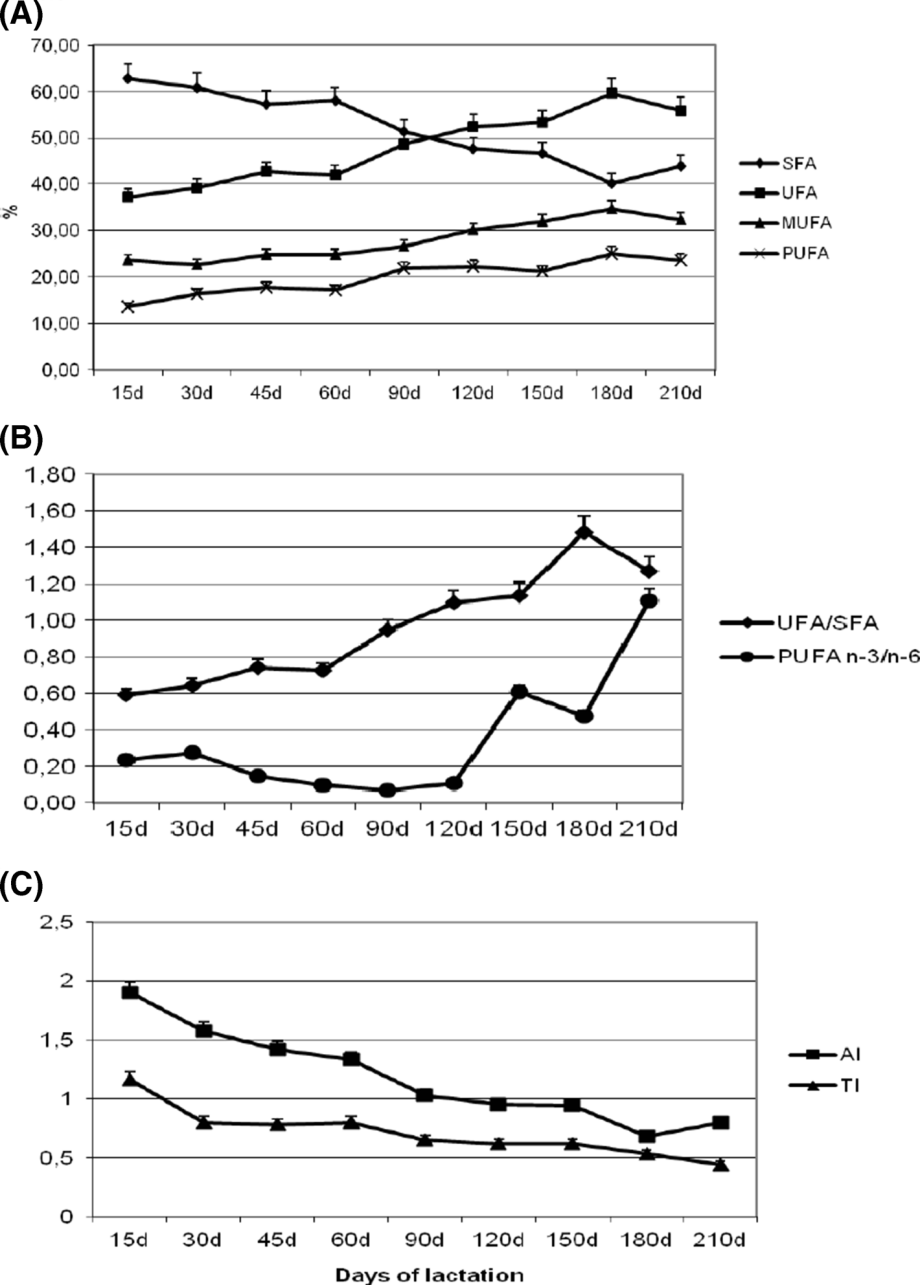
Variations of fatty acid composition and indices for human health of milk during the lactation period: (A) saturated (SFA), unsaturated (UFA), monounsaturated (MUFA) and polyunsaturated fatty acids (PUFA); (B) UFA/SFA and PUFA n-3/n-6 fatty acids ratios; (C) atherogenic (AI) and thrombogenic (TI) indices (mean ± SD).

The SFA content in the milk decreased significantly (P < 0.01) during the lactation period (−33.9%), from an average mean of 62.88% at day 15 to 41.54% at day 180 after foaling and then decreased slightly at day 210 (Figure 1, Table 3a).

**Table 3:** Statistical differences in (a) SFA (saturated fatty acids), (b) C8:0, (c) C10:0, (d) C12:0, (e) C14:0, (f) C16:0 and (g) UFA (unsaturated fatty acids)/SFA ratio of milk in relation to the lactation stage of jennies (*: P < 0.05; **: P < 0.01; ns: not significant)

**(a) SFA**												**(c) C10:0**									
**(b) C8:0**		**15d**	**30d**	**45d**	**60d**	**90d**	**120d**	**150d**	**180d**	**210d**	**(d) C12:0**		**15d**	**30d**	**45d**	**60d**	**90d**	**120d**	**150d**	**180d**	**210d**
	**15d**		ns	ns	ns	**	**	**	**	**		**15d**		ns	ns	ns	ns	**	**	**	**
	**30d**	ns		ns	ns	**	**	**	**	**		**30d**	ns		ns	ns	ns	**	**	**	**
	**45d**	ns	ns		ns	ns	**	**	**	**		**45d**	ns	ns		ns	ns	**	**	**	**
	**60d**	*	ns	ns		ns	**	**	**	**		**60d**	ns	ns	ns			**	**	**	**
	**90d**	ns	ns	ns	ns		ns	ns	**	*		**90d**	*	ns	ns	ns		**	*	**	*
	**120d**	ns	ns	ns	**	**		ns	*	ns		**120d**	**	ns	*	ns	ns		ns	ns	ns
	**150d**	ns	**	**	**	*	ns		ns	ns		**150d**	**	*	**	*	ns	ns		ns	ns
	**180d**	ns	**	**	**	**	ns	ns		ns		**180d**	**	**	**	**	*	ns	ns		ns
	**210d**	ns	**	*	**	*	ns	ns	ns			**210d**	**	*	**	**	ns	ns	ns	ns	
**(e) C14:0**												**(g) UFA/SFA**									
		**15d**	**30d**	**45d**	**60d**	**90d**	**120d**	**150d**	**180d**	**210d**			**15d**	**30d**	**45d**	**60d**	**90d**	**120d**	**150d**	**180d**	**210d**
**(f) C16:0**	**15d**		ns	ns	*	**	**	**	**	**		**15d**		ns	ns	ns	*	**	**	**	**
	**30d**	**		ns	ns	*	*	**	**	**		**30d**			ns	ns	ns	**	**	**	**
	**45d**	ns	ns		ns	ns	ns	**	**	**		**45d**				ns	ns	**	**	**	**
	**60d**	**	ns	ns		ns	ns	*	**	*		**60d**					ns	**	**	**	**
	**90d**	**	ns	ns	ns		ns	ns	*	ns		**90d**						ns	ns	**	ns
	**120d**	**	ns	ns	ns	ns		ns	ns	ns		**120d**							ns	*	ns
	**150d**	ns	**	ns	ns	**	ns		ns	ns		**150d**								ns	ns
	**180d**	*	ns	ns	ns	ns	ns	ns		ns		**180d**									ns
	**210d**	**	ns	ns	ns	ns	ns	ns	ns			**210d**									

Among the SFA, palmitic acid was observed to be the most concentrated (C16:0 = 19.94% average, data not presented). Lower concentration levels were observed for capric (C10:0 = 10.78% average), lauric (C12:0 = 8.78% average), myristic (C14:0 = 6.98% average) and caprylic (C8:0=6.20%) acids, with the lowest level observed for stearic acid (C18:0=1.82% average) (data not presented).

Among the fatty acids found at concentrations greater than 1%, palmitic acid (C16:0) exhibited a significant decrease in concentration at day 30 and day 90 (Tables 4, 3f). The myristic acid (C14:0) concentration decreased significantly from day 15 to day 120, day 150 and day 180 (Tables 4, 3e). Lauric acid (C12:0) exhibited a significant decrease in concentration from day 15 to day 180 (Tables 4, 3d). Caprylic acid (C8:0) showed a slight increase in concentration at day 30, followed by a significant decrease until reaching 3.79% at day 180 (Tables 4, 3b). Capric acid (C10:0), after a slight increase between 15 and 30 days, declined in concentration significantly to 5.86% at day 180 (Tables 4, 3c). The stearic acid concentration did not vary significantly over time (Table 4).

**Table 4:** Saturated, monounsaturated, polyunsaturated fatty acid profile (% of fatty acids) and desaturase index during the lactation period (210days)

	**LACTATION STAGE**										**Effect of Lactation stage**^**1**^
		**15 d**	**30 d**	**45 d**	**60 d**	**90 d**	**120 d**	**150 d**	**180 d**	**210 d**	
*Saturated Fatty Acids (SFA)*											
** Butyric Acid**	**C**_**4:0**_	0.51	0.61	0.28	0.51	0.4	0.57	0.59	0.15	0.23	*
** Caproic Acid**	**C**_**6:0**_	0.52	0.72	0.58	0.72	0.71	0.64	0.61	0.37	0.59	*
** Heptanoic Acid**	**C**_**7:0**_	0.02	0.01	0.02	0.01	0.01	0.03	0.02	0.01	0.03	ns
** Caprylic Acid**	**C**_**8:0**_	5.30	6.76	6.13	6.87	6.07	4.57	4.39	3.79	4.73	*
** Nonanoic Acid**	**C**_**9:0**_	0.04	0.03	0.03	0.03	0.03	0.03	0.03	0.04	0.03	ns
** Capric acid**	**C**_**10:0**_	12.01	13.46	11.75	11.93	9.82	7.62	7.19	5.82	6.66	**
** Undecanoic acid**	**C**_**11:0**_	0.03	0.03	0.02	0.02	0.02	0.02	0.02	0.02	0.01	ns
** Lauric acid**	**C**_**12:0**_	10.82	10.87	9.47	9.27	7.55	6.42	5.84	4.44	5.32	**
** Tridecanoic acid**	**C**_**13:0**_	0.04	0.05	0.06	0.06	0.05	0.04	0.03	0.03	0.04	ns
** Myristic Acid**	**C**_**14:0**_	8.66	7.80	7.20	6.82	5.79	5.60	5.08	3.91	4.95	**
** Pentadecanoic Acid**	**C**_**15:0**_	0.22	0.23	0.23	0.23	0.24	0.23	0.23	0.20	0.22	ns
** Palmitic Acid**	**C**_**16:0**_	22.37	18.33	19.56	19.06	18.53	19.61	20.83	19.82	19.47	*
** Margaric acid**	**C**_**17:0**_	0.22	0.22	0.24	0.24	0.26	0.26	0.24	0.22	0.22	ns
** Stearic Acid**	**C**_**18:0**_	1.88	1.48	1.64	1.99	1.68	1.75	1.52	1.49	1.44	ns
** Arachidic Acid**	**C**_**20:0**_	0.10	0.09	0.08	0.09	0.09	0.10	0.08	0.09	0.07	ns
** Heneicosanoic Acid**	**C**_**21:0**_	0.03	0.04	0.02	0.04	0.03	0.02	0.02	0.03	0.02	ns
** Behenic Acid**	**C**_**22:0**_	0.04	0.10	0.15	0.06	0.07	0.19	0.15	0.05	0.04	ns
** Lignoceric Acid**	**C**_**24:0**_	0.06	0.11	0.07	0.08	0.09	0.09	0.06	0.08	0.06	ns
*Monounsaturated Fatty Acids (MUFA)*											
** Decenoic Acid**	**C**_**10:1**_	1.38	1.64	1.25	1.36	1.08	0.91	0.84	0.76	1.20	*
** Dodecenoic Acid**	**C**_**12:1**_	0.17	0.18	0.14	0.15	0.11	0.11	0.10	0.08	0.13	ns
** Tridecenoic acid**	**C**_**13:1**_	0.04	0.04	0.04	0.04	0.04	0.04	0.03	0.09	0.05	ns
** Myristoleic Acid**	**C**_**14:1**_	0.38	0.34	0.29	0.26	0.21	0.26	0.25	0.21	0.32	*
** Pentadecenoic acid**	**C**_**15:1**_	0.07	0.08	0.08	0.06	0.07	0.07	0.06	0.06	0.08	ns
** Palmitoleic Acid**	**C**_**16:1**_	3.02	2.79	2.98	2.36	2.28	3.22	4.23	4.48	5.67	*
** Heptadecenoic Acid**	**C**_**17:1**_	0.30	0.29	0.33	0.33	0.31	0.34	0.38	0.31	0.40	ns
** Oleic Acid**	**C**_**18:1 n-9**_	17.55	16.65	18.62	19.36	21.29	23.74	25.05	27.57	23.73	**
** Gadoleic Acid**	**C**_**20:1 n-9**_	0.25	0.31	0.37	0.42	0.45	0.49	0.38	0.39	0.34	ns
** Erucic Acid**	**C**_**22:1 n-9**_	0.40	0.31	0.64	0.44	0.75	0.81	0.59	0.46	0.37	ns
** Nervonic Acid**	**C**_**24:1**_	0.01	0.02	0.03	0.01	0.02	0.01	0.01	0.05	0.02	ns
*Polyunsaturated Fatty Acids (PUFA)*											
** Linoleic Acid**	**C**_**18:2 n-6**_	8.83	9.76	10.76	11.20	15.15	14.64	12.93	17.20	11.03	**
** Linolenic Acid**	**C**_**18:3 n-3**_	3.85	5.71	5.63	4.81	5.20	5.99	6.96	6.36	11.17	**
** Stearidonic Acid**	**C**_**18:4 n-3**_	0.01	0.02	0.02	0.02	0.02	0.03	0.03	0.03	0.03	ns
** Eicosadienoic Acid**	**C**_**20:2 n-6**_	0.22	0.20	0.30	0.29	0.36	0.34	0.23	0.24	0.19	ns
** Eicosatrienoic Acid**	**C**_**20:3 n-3**_	0.03	0.03	0.03	0.03	0.04	0.03	0.03	0.04	0.03	ns
** Arachidonic Acid**	**C**_**20:4 n-6**_	0.08	0.13	0.08	0.08	0.08	0.08	0.09	0.10	0.11	ns
** Eicosatetraenoic Acid**	**C**_**20:4 n-3**_	0.10	0.10	0.14	0.14	0.13	0.13	0.16	0.14	0.24	ns
** Eicosapentaenoic Acid**	**C**_**20:5 n-3**_	0.23	0.30	0.56	0.48	0.65	0.71	0.49	0.55	0.51	ns
** Docosadienoic Acid**	**C**_**22:2 n-6**_	0.05	0.06	0.08	0.06	0.10	0.11	0.12	0.10	0.09	ns
** Docosatrienoic Acid**	**C**_**22:3 n-3**_	0.00	0.01	0.00	0.02	0.01	0.01	0.01	0.01	0.01	ns
** Docosatetraenoic Acid**	**C**_**22:4 n-6**_	0.05	0.05	0.05	0.04	0.07	0.09	0.03	0.05	0.03	ns
** Docosapentaenoic Acid**	**C**_**22:5 n-3**_	0.04	0.03	0.07	0.03	0.03	0.04	0.04	0.13	0.05	ns
** Docosahesaenoic Acid**	**C**_**22:6 n-3**_	0.04	0.01	0.03	0.02	0.02	0.02	0.02	0.03	0.02	ns
*Δ9-desaturase ratios*^*a*^											
	***cis*-9 14:1**	0.04	0.04	0.04	0.04	0.03	0.04	0.05	0.06	0.06	*
	***cis*-9 16:1**	0.12	0.13	0.13	0.11	0.11	0.14	0.16	0.17	0.22	**
	***cis*-9 18:1**	0.90	0.92	0.92	0.91	0.93	0.93	0.94	0.95	0.94	**

^a^ Ratio of the Δ9-desaturase product divided by the sum of Δ9-desaturase product and product as described in Materials and Methods.

^1^ns (non-significant): P>0.05; * P<0.05; ** P<0.01.

### Unsaturated fatty acid

The overall unsaturated fatty acid (UFA) content of the milk was 48.02 ± 2.97% (Table 2) and increased during the lactation period (Figure 1A). The mean UFA/SFA ratio was 0.92, which underwent a significant increase over time, reaching a value greater than 1% at day 120 and a peak of 1.50% at day 180 (Figure 1B, Table 3g).

The overall MUFA content was 28.00 ± 3.91% (Table 2). The stage of lactation affected (0.05 > P < 0.01) the MUFA content. The total MUFA increased (P<0.01) during lac-tation from 23.58% at day 15 to 34.40% at day 180 and then decreased slightly at day 210 (Figure 1A, Table 5a). The individual MUFA profiles are reported in Table 4. Oleic acid (C18:1 n-9) was the most representative of MUFA (21.50%, average), with a significant (Table 5g) increase at day 90 and maximum level at day 180 (27.57%). The average palmitoleic acid (C16:1) content was 3.45%. It decreased from early lactation to day 90, then increased significantly (Table 5e), reaching its maximum level at day 210.

**Table 5:** Statistical differences in (a) MUFA (monounsaturated fatty acids), (b) C10:1, (c) C14:1, (d) C14:1/C14:0, (e) C16:1, (f) C16:1/C16:0, (g) C18:1 and (h) C18:1/C18:0 of milk in relation to the lactation stage of jennies (*: P < 0.05; **: P < 0.01; ns: not significant)

**(a) MUFA**												**(c) C14:1**									
**(b) C10:1**		**15d**	**30d**	**45d**	**60d**	**90d**	**120d**	**150d**	**180d**	**210d**	**(d) C14:1/ C14:0**		**15d**	**30d**	**45d**	**60d**	**90d**	**120d**	**150d**	**180d**	**210d**
	**15d**		ns	ns	ns	ns	*	**	**	**		**15d**		ns	ns	*	**	*	*	**	ns
	**30d**	ns		ns	ns	ns	**	**	**	**		**30d**	ns		ns	ns	**	ns	ns	*	ns
	**45d**	ns	ns		ns	ns	ns	**	**	**		**45d**	ns	ns		ns	ns	ns	ns	ns	ns
	**60d**	ns	ns	ns		ns	ns	**	**	**		**60d**	ns	ns	ns		ns	ns	ns	ns	ns
	**90d**	ns	**	ns	ns		ns	*	**	*		**90d**	ns	ns	ns	ns		ns	ns	ns	*
	**120d**	**	**	**	**	**		ns	ns	ns		**120d**	ns	ns	ns	ns	ns		ns	ns	ns
	**150d**	**	**	**	**	*	ns		ns	ns		**150d**	ns	ns	ns	*	*	ns		ns	ns
	**180d**	**	**	**	**	**	ns	ns		ns		**180d**	ns	ns	ns	*	*	ns	ns		*
	**210d**	**	**	**	**	*	ns	ns	ns			**210d**	**	**	**	**	**	**	**	*	
	**(e) C16:1**											**(g) C18:1**									
		**15d**	**30d**	**45d**	**60d**	**90d**	**120d**	**150d**	**180d**	**210d**	**(h) C18:1/ C18:0**		**15d**	**30d**	**45d**	**60d**	**90d**	**120d**	**150d**	**180d**	**210d**
**(f) C16:1/ C16:0**	**15d**		ns	ns	ns	ns	ns	ns	ns	**		**15d**		ns	ns	ns	ns	**	**	**	**
	**30d**	ns		ns	ns	ns	ns	ns	ns	**		**30d**	ns		ns	ns	*	**	**	**	**
	**45d**	ns	ns		ns	ns	ns	ns	ns	**		**45d**	ns	ns		ns	ns	*	**	**	**
	**60d**	ns	ns	ns		ns	ns	ns	*	**		**60d**	*	ns	ns		ns	*	**	**	*
	**90d**	ns	ns	ns	ns		ns	*	*	**		**90d**	ns	ns	ns	ns		ns	ns	**	ns
	**120d**	ns	ns	ns	ns	ns		ns	ns	**		**120d**	*	ns	ns	ns	ns		ns	ns	ns
	**150d**	ns	ns	ns	*	*	ns		ns	ns		**150d**	**	*	*	**	ns	ns		ns	ns
	**180d**	ns	ns	ns	*	*	ns	ns		ns		**180d**	**	**	*	**	ns	ns	ns		ns
	**210d**	**	**	**	**	**	**	**	*			**210d**	**	*	*	**	ns	ns	ns	ns	

Among the MUFAs found at a concentration greater than 1%, the content of decenoic acid (C10:1=1.16%, average) decreased significantly during lactation (Tables 4, 5b). The miristoleic acid (C14:1) content exhibited a decreasing trend, from day 15 to day 180, and then increased at day 210 (Tables 4, 5c).

With respect to milk fatty acid pairs representing product/substrate ratios and related to the Δ^9^-desaturase system (Table 2), a higher desaturase index was observed (P < 0.01) for *cis*-9 18:1 (0.93, average) than for *cis*-9 16:1 (0.14, average) and *cis*-9 14:1 (0.04, average). The stage of lactation affected (P < 0.01) the Δ^9^-desaturase ratios (Table 4), with a significant increase mainly in *cis*-9 18:1/18:0 (Table 5h) and *cis*-9 16:1/16:0 (Table 5f) as lactation progressed.

Among the observed UFAs, the contents of PUFAs were lower (P < 0.05) than those of MUFAs (Table 2). The total PUFA contents were affected by the stage of lactation (P < 0.01), increasing (P < 0.01) from day 15 to day 45 and then until day 180 (Figure 1A, Table 6a).

**Table 6:** Statistical differences in (a) PUFA (polyunsaturated fatty acids), (b) PUFA n3/n6 ratio, (c) C18:2, (d) C18:3, (e) AI (atherogenic index) and (f) TI (thrombogenic index) of milk in relation to the lactation stage of jennies (*: P < 0.05; **: P < 0.01; ns: not significant)

**(a) PUFA**												**(c) C18:2**									
		**15d**	**30d**	**45d**	**60d**	**90d**	**120d**	**150d**	**180d**	**210d**	(d) **C18:3**		**15d**	**30d**	**45d**	**60d**	**90d**	**120d**	**150d**	**180d**	**210d**
**(b) n3/n6**	**15d**		ns	**	*	**	**	**	**	**		**15d**		ns	ns	ns	**	**	**	**	**
	**30d**	ns		ns	ns	**	**	**	**	**		**30d**	ns		ns	ns	**	**	*	**	ns
	**45d**	ns	ns		ns	**	**	*	**	**		**45d**	ns	ns		ns	**	**	ns	**	ns
	**60d**	ns	ns	ns		**	**	**	**	**		**60d**	ns	ns	ns		**	*	ns	**	ns
	**90d**	ns	ns	ns	ns		ns	ns	*	ns		**90d**	ns	ns	ns	ns		ns	ns	ns	**
	**120d**	ns	ns	ns	ns	ns		ns	ns	ns		**120d**	ns	ns	ns	ns	ns		ns	ns	ns
	**150d**	ns	ns	ns	ns	ns	ns		**	ns		**150d**	*	ns	ns	ns	ns	ns		ns	ns
	**180d**	ns	ns	ns	ns	ns	ns	ns		ns		**180d**	ns	ns	ns	ns	ns	ns	ns		**
	**210d**	**	**	ns	ns	ns	ns	**	**			**210d**	**	**	**	**	**	**	**	**	
	**(e) AI**																				
		**15d**	**30d**	**45d**	**60d**	**90d**	**120d**	**150d**	**180d**	**210d**											
**(f) TI**	**15d**		ns	*	**	**	**	**	**	**											
	**30d**	**		ns	ns	**	**	**	**	**											
	**45d**	**	ns		ns	**	**	**	**	**											
	**60d**	**	ns	ns		ns	*	*	**	**											
	**90d**	**	ns	ns	ns		ns	ns	ns	ns											
	**120d**	**	ns	ns	ns	ns		ns	ns	ns											
	**150d**	**	ns	ns	ns	ns	ns		ns	ns											
	**180d**	**	**	**	**	ns	ns	ns		ns											
	**210d**	**	**	**	**	**	ns	**	ns												

The PUFA n-6 content of milk fat was higher than that of the PUFA n-3 series (Table 2); both increased during lactation. The PUFA n-6 content underwent a significant increase from day 15 (9.19%) to day 90 (15.70%; P < 0.01), reached a maximum value at day 180 (17.65%; P < 0.01) and then decreased at day 210 (11.44%) (data not provided). The PUFA n-3 content increased during the lactation period, reaching a peak at day 210 (12.08%; P < 0.01).

Among the PUFA of the n-6 series, the most abundant acid was linoleic acid (C18:2 n-6, LA=18.38%, average). The LA content was 8.83% at day 15, with a significant increase at day 90 and the highest level at day 180 (17.20%), which was followed by a marked decrease at day 210 (Tables 4, 6c).

Among the PUFAs of the n-3 series, alpha-linolenic acid (ALA, C18:3 n-3) was the most represented (6.19%, average). The ALA content underwent a significant increase from 3.85% at day 15 to 6.36% at day 150 and reached a maximum level at day 210 (11.17%) (Tables 4, 6d).

Among the minor PUFAs, eicosadienoic (C20:2 n-6), arachidonic (AA, C20:4 n-6), eicosapentanoic (EPA, C20:5 n-3), eicosatetranoic (ETA, C20:4 n-3) and docosahesaenoic (DHA, C22:6 n-3) acid were present at mean concentrations of 0.26%, 0.09%, 0.50%, 0.14% and 0.02%, respectively (data not presented); no significant variations were observed during the lactation period (Table 4).

The overall PUFA n-3/n-6 ratio was 0.59 (Table 2), with a significant increase from early lactation to a maximum value at day 210 (Figure 1B, Table 6b).

The means of the AI and TI were 1.16 and 0.70, respectively (data not presented) and were significantly (P < 0.01) affected by the lactation stage. The AI decreased markedly from day 15 to day 45, reaching values less than 1 at day 120 and a minimum at day 180 (0.68) (Figure 1c, Table 6e). The TI decreased over time showing a significant drop at day 30 until reaching the lowest value of 0.44 at day 210 of lactation (Figure 1C, Table 6f).

## Discussion

The observed low average milk fat content is consistent with that reported in the literature for donkey’s milk [24,25] and lower than that reported for mares [26], cows and humans [27].

The fat content was affected by the stage of lactation, showing a decreasing trend, which is in agreement with the results observed for the Ragusana breed [10,28], Litoral-Dinaric donkeys [25], mares [9,29,30] and cows [31].

The low fat content of milk and its variations during the lactation period corresponded to a low energetic value with the same trend during lactation. The gross energy content of the milk was similar to that recorded by Salimei et al. [11]. Compared with the energetic value of milk from different species, that of donkey milk is slightly lower than that of mare milk [29,32] and lower than that of cow and human milk [27]. This finding could be a limiting factor in its use in infant nutrition within a diet exclusively based on milk. Thus, it has been suggested that an appropriate modification in the composition of donkey’s milk be made for infant feeding [33] by adding medium-chain triglycerides or sunflower oil [34,35]. However, the low fat content and energetic value of this milk suggests its potential use in hypocaloric human diets.

The decreasing energy content of donkey’s milk during lactation agrees with the trend observed in mares [29,32], although Salimei et al. [11] did not observe any considerable variation in fat content or energy value during lactation, which is likely due to different experimental conditions.

The SFAs were the most representative fatty acids, in agreement with the results of other studies [36,37] and comparable to the composition of mare [38] and human milk [27]; however, its content were observed to be lower than those observed in ruminant milk [5]. The short-chain fatty acids and medium-chain fatty acids contents as in mares, were observed to be lower than those observed in cow’s milk and higher than those observed in human milk [26,39]. The high fatty acid content < C16 content observed in this study, with respect to other monogastric species [9], agrees with the results from equine studies and could be attributed to their synthesis from acetate and 3-hydroxybutyrate, as observed in ruminants, and not from glucose as occurs in monogastrics [9,40]. Furthermore, instead of ruminants, fatty acid *de novo* synthesis in equines includes parts of C18 next to C4 and C14 and parts of C16 fatty acids [40].

Among the observed SFA, butyric (C4:0) and caproic (C6:0) acids were observed in concentrations less than 1%, as has been observed in mares [26]. The average amounts of caprylic (C8:0), capric (C10:0) and lauric (C12:0) acids were lower than those reported for Ragusana jennies (12.80%, 18.65%, and 10.67%, respectively; [41]); this finding is likely related to differences in breed and/or body conditions. The caprylic (C8:0) acid content was higher compared with that reported for mares (3.1%), cows (1.3%) and human milk (traces) [40]. Among the observed SFAs, palmitic acid (C16:0) was observed at the highest concentration, in agreement with the results reported in other related studies [28,37] and in studies on mares [26]; however, the content was less than that of cow and human milk [26,39].

During the early lactation period (15–30days), the milk was observed to contain more C4:0 to C14:0 than during the middle (day 120) and late (day 210) lactation periods, most likely due to an increased synthesis of C4 to C14 fatty acids in the mammary gland during the early lactation period, as has been observed in cows [42].

Long-chain fatty acids such as stearic acid (C18:0) were observed in modest amounts, consistent with the results reported for other breeds [37,41] as well as for mares (1.55%) [9,43], while it has been recorded at higher levels in cow and human milk (7-13%) [26,27,37]. As in equines, the low stearic acid content could be attributed to a low stearic acid content in the animals’ diets and to a high activity of Δ^9^-desaturase on the conversion of stearic acid (C18:0) into oleic acid (C18:1), which is further enhanced by a high palmitoleic acid (C16:1) content [26], as was observed in this study.

The SFA content decreased during lactation. A higher decrease was observed for lauric (C12:0, -59%) and myristic (C14:0, -54.5%) acids and a lower decrease for palmitic acid (C16:0, -17%), likely due to a decrease in the concentration of the precursors of *de novo* synthesis. A decrease in the short- and medium-chain fatty acid contents during lactation has also been reported in mares [9,44], while small changes in the SFA content have been observed in human milk [45]; additionally, increases in these fatty acid contents have been observed in cow’s milk [46].

From a nutritional standpoint, it has been reported that C18 SFA has a neutral health effect, while C4 to C10 SFAs have positive effects and C12 to C16 SFAs have negative health effects [47]. In particular, SFAs C14 - C16 are considered dangerous because they are associated with high serum LDL-cholesterol concentrations in human subjects [48]. Medium-chain fatty acids are characterised by better absorption than long-chain fatty acids [49]. Furthermore, it has been suggested that equids’ milk is more digestible than cow’s milk, according to experiments performed on rats, and allows for faster evacuation of the stomach [26].

The UFA content, in agreement with other studies (50.69%) [13], was similar to that of mare’s and human milk but higher than that reported for ruminant’s milk (23-32%) [27,41].

The UFA/SFA ratio is comparable to mare’s milk [27] and higher than that recorded in the Ragusana donkey (0.48) and that reported for ruminant’s milk (0.26-0.41) [41,47].

The MUFA content and oleic (C18:1 n-9) and palmitoleic (C16:1 n-7) acid contents were higher than those observed in donkey’s milk by other researchers [11,41], likely due to different experimental conditions; these contents were observed to be similar to those reported for mares but lower than those in human milk (C18:1, 46%) [30].

The MUFA content increased during the lactation period, whereas Chiofalo et al. [28] did not find significant variability throughout lactation in the Ragusana breed, most likely due to different experimental conditions.

In this study, the amount of desaturation, calculated from the ratio of Δ^9^-desaturase product/sum of Δ^9^-desaturase product and substrate, was relatively high for C18:1 and C16:1 and increased as lactation progressed, reaching higher values at 150 to 210 days of lactation. This result may explain the observed high value of these MUFAs in milk and their increased trend during lactation. Moreover, it has been reported that preformed fatty acids, such as oleic acid, could be derived from mobilisation from adipose tissue or from absorption from the digestive tract, with an increase being observed for a forage diet [26,44], which likely occurred in this study for the asses fed on pasture.

It has been reported that in the human diet the high relative percentage of MUFA has beneficial effects with respect to arterial disease, i.e., it lowers plasma LDL cholesterol and total cholesterol as well as the fibrinolytic activity of circulating plasma by modifying vascular endothelial physiology [50], while the intake of SFA is a risk factor in contracting coronary heart disease [51].

Among PUFAs and EFAs, LA (C18:2 n-6) and ALA (C18:3 n-3) acid were the most representative of PUFA n-6 and PUFA n-3, respectively, exhibiting higher values than those of the Ragusana breed [41] and comparable to the range recorded in mares [30] but higher than that reported in ruminant’s milk [47]. Furthermore, the n-3/n-6 fatty acid ratio was higher than that recorded in Ragusana asses (0.19) [52] and higher than that reported in humans (0.012) or in cows (0.28) [37]. As in equines, according to other researchers [19,30], the reasons for this difference could be related to the amounts of these acids in the animals’ diets and the absence of biohydrogenation of fatty acids in the digestive tract before absorption, unlike what occurs in ruminants [53].

In humans, LA has been reported to play a role in the prevention of gastric ulcers. It is a precursor of prostaglandin E that increases with stimulation by linoleic acid [54]. Prostaglandins are involved in the prevention of gastric ulcers through a rise in mucosal protective factors; this prevention is important in foals, which have a certain incidence of gastric ulcers [55].

In agreement with the results of Gastaldi et al. [37], the percentage of ALA was higher compared with that of human’s (1.14%) and cow’s milk (0.48%) whereas the LA content was similar to that reported for human milk.

The ALA content increased during the lactation period, consistent with the results observed for mares [56], and the maximum level was achieved late in the lactation period. This result reflects the higher PUFA n-3/n-6 ratio at the 7^th ^month of lactation (day 210). This higher ratio may be explained by the increase in pasture feeding by jennies on richer grass during the late lactation period. Grass is rich in ALA content, and no biohydrogenation occurs before absorption [30]. Furthermore, higher ALA contents have been reported in milk from asses [57] and mares [26] fed on herbage rather than hay.

AI and TI, which indicate the healthfulness of milk with respect to fatty acid content and their potential to prevent or cause atherosclerosis and thrombosis, were observed to be present in lower quantities than in cow’s milk (2.51 and 1.86, respectively) [58].

It should be noted that LA is the precursor of PUFA n-6, and AA (C20:4 n-6) and ALA are the precursors of PUFA n-3, EPA (C20:5 n-3) and DHA (C22:6 n-3) acids. AA and EPA/DHA are substrates for the formation of eicosanoids [59]. Prostaglandins and leukotrienes are important in controlling several cell activities [60], and DHA is important in the development of the central nervous system [61]. The role of PUFA is important in the development of the neonatal brain as well as in the development of the retina and cognitive functions [62-64]. The high content of n-3 fatty acids in donkey milk could have a significant effect on the development of the neural system, vision and infant growth.

Among the minor PUFA observed in this study, small amounts of EPA, DHA and AA were observed, consistent with other studies on donkey’s milk [11,37,41] and mare’s milk [26]. The percentage of EPA was higher compared with human milk (0.27%); in contrast, the DHA and AA contents were lower (0.40 and 0.59%) [37]. The reasons for this result are likely due to the different elongation and desaturation processes in mammary glands. A higher AA/EPA ratio in breast milk from the mothers of atopic infants compared with non-atopic infants [65] and the relationship between lower levels of EPA and the early development of atopic disease have been reported. The low AA/EPA ratio observed in this study (0.18), with respect to those reported for human’s and cow’s milk (29.5 and 3.67) [37], suggests that donkey milk could be used in childhood nutrition to prevent the risk of developing atopical diseases. Moreover, donkey’s milk could be useful in pre-term infants’ diets because premature infants seem able to form AA from linoleic acid and EPA and DHA from α-linolenic acid [66,67]. The positive effects of donkey’s milk in infant nutrition has been demonstrated in the treatment of multiple food intolerances [34,68,69] and in selected cases of cow’s milk allergies [7,70].

The high recorded amounts of PUFAs in donkey milk suggest that this milk may also be used adult human diets. From a nutritional point of view, it has been reported that the rapid changes in the diet of the Western industrialised society, mainly over the last 150 years with respect to the ancestor-constituted genetic profile, involve a particular increase in saturated fats and n-6 fatty acids and a decrease in n-3 fatty acids [71-74]. The levels of long-chain n-6 to n-3 (mainly LA compared with ALA) fatty acids are important, particularly n-3 fatty acids [75], in maintaining cardiovascular health [76,77] and they influence the ratios of ensuing eicosanoids and metabolic functions [59]. The dietary balance of the ratios of n-6 to n-3 PUFA affects the regulation of metabolic functions [78] and the development of metabolic syndrome, including lipid profile and adiposity, insulin sensitivity and inflammation [79,80]. It has been estimated that the ratio of n-6 to n-3 fatty acids in the Western diet is 15-20/1, and more n-3 than n-6 fatty acids have been suggested, with a ratio that tends towards 1:1 [81].

In this study, the PUFA n-6 to n-3 ratio and the LA/ALA ratio, in particular, were approximately 2:1, with a lower value (< 1) during the 7^th^ month of lactation due to a high increase in the ALA content, potentially suggesting the more optimal use of milk collected during this period.

In addition to the benefits related to coronary health disease, the beneficial health effects of n-3 fatty acids include those related to inflammatory disease, such as rheumatoid arthritis [82], dermatitis [83,84], cancer [85], depression and dementia, and may be potentially used to treat late-onset Alzheimer’s disease [86,87].

Long-chain PUFA n-3 has been shown to induce immunomodulatory activity on natural and acquired immunity [88,89] through the synthesis of lipid mediators (pros-taglandins, leukotrienes), peptide mediators (cytokines), reactive oxygen species (superoxide), and enzymes. The metabolites of n-3 fatty acids are less inflammatory than those of n-6 (in particular, AA). The eicosanoid derived from AA, i.e, prostaglandin E2 and leukotriene B4, promote atopic inflammation; in contrast, PUFA n-3 and their derived eicosanoid influence AA metabolism, promoting anti-inflammatory properties [90,91]. The high PUFA n-3 content in donkey’s milk could have a functional effect on the immunological system. In our study on an in vitro model of human peripheral blood mononuclear cells [6,92], donkey milk exhibited the ability to induce IgG secretion and the release of interleukins (IL-12, IL-1β, IL-10) and TNF-α - important to the immunotreatment of immune-related disease - and a high release of nitric oxide (NO), a potent promoter in the prevention of atherosclerosis. Indeed, NO is a strong vasodilator of terminal vessels, improves blood flow, and is an effective antimicrobial agent in the development of atherosclerosis [93]. In combination, these results support the concept that donkey’s milk, likely due to its high PUFA n-3 content, can prevent atherosclerosis via the production of NO and, at moderate intake level (200 mL/d; [94]), can up-regulate the immune response in elderly hosts.

## Conclusions

Donkey milk is characterised by low fat and energetic values, a high PUFA content composed mainly of ALA (18:3 n-3) and LA (18:2 n-6), a low ratio of n-6 to n-3 fatty acids or LA/ALA ratio and advantageous values of AI and TI. Among minor PUFA, DHA, EPA, and AA were present in minute amounts.

The fat and energetic values decreased during lactation. The fatty acids patterns were influenced by the lactation stage, showing a decrease in SFA content and an increase in UFA content, with higher values being recorded during the late lactation period. During this period, lower values of the n-6/n-3 ratio (<1) were also observed. The higher PUFA n-3 content and lower PUFA n-6/n-3 and AA/EPA ratios of donkey’s milk with respect to cow’s milk may be of particular interest in adopting donkey’s milk for infant nutrition. Considering the low lipid and energetic value content, donkey’s milk could be used within well-balanced and integrated hypocaloric diets. The recorded high PUFA content and low n-6/n-3 or LA/ALA ratios in donkey milk could make it interesting for adult human nutrition.

## Competing interests

The authors declare that they have no competing interests.

## Authors’ contributions

All authors have read and approved the final manuscript. AGD is the leading scientist in this study. AGD and GM contributed and approved the final manuscript. They conceived and carried out the study design, participated in the chemical analysis of milk lipids, as well as analyzing the data, performing the statistical analysis and drafting the manuscript.
